# Humor in radiological breast cancer screening: a way of improving patient service?

**DOI:** 10.1186/s40644-022-00493-z

**Published:** 2022-10-08

**Authors:** Elisabeth Sartoretti, Thomas Sartoretti, Dow Mu Koh, Sabine Sartoretti-Schefer, Sebastian Kos, Romana Goette, Ricardo Donners, Robyn Benz, Johannes M. Froehlich, Simon Matoori, Peter Dubsky, Tino Plümecke, Rosemarie Forstner, Willibald Ruch, Matthias Meissnitzer, Klaus Hergan, Selina Largiader, Andreas Gutzeit

**Affiliations:** 1 Faculty of Medicine, University of Zürich, Pestalozzistrasse 3, 8032 Zürich, Switzerland; 2 Present Address: Department of Health Sciences and Medicine, University of Lucerne, Frohburgstrasse 3, Postfach 4466, 6002 Luzern, Switzerland; 3 Cancer Research UK Clinical Magnetic Resonance Research Group, Institute of Cancer Research, Downs Road, Sutton, Surrey, SM2 5PT UK; 4 Department of Radiology, Cantonal Hospital Winterthur, Brauerstrasse 15, 8401 Winterthur, Switzerland; 5 Institute of Radiology and Nuclear Medicine and Breast Center St. Anna, Hirslanden Klinik St. Anna, St. Anna- Strasse 32, 6006 Lucerne, Switzerland; 6 Department of Radiology, University Hospital Basel, University of Basel, Süotaöstrasse 21/ Petersgraben 4, 4031 Basel, Switzerland; 7 Department of Diagnostic, Interventional and Pediatric Radiology, Inselspital, University of Bern, Freiburgstrasse 10, 3010 Bern, Switzerland; 8 Clinical Research Group, Klus Apotheke Zürich, Hegibachstrasse 102, 8032 Zürich, Switzerland; 9 Faculté de Pharmacie, Université de Montréal, Pavillon Jean- Coutu 2940, chemin de Polythechnique, Montréal, Québec, H3T 1J4 Canada; 10 Breast Center, Hirslanden Clinic St Anna, St. Anna- Strasse 32, 6006 Lucerne, Switzerland; 11 Institute of Sociology, University of Freiburg, Freiburg, Germany; 12 Department of Radiology, Paracelsus Medical University, Müllner- Hauptstrasse 48, 5020 Salzburg, Austria; 13 Department of Psychology, University of Zurich, Binzmühlestrasse 14, Box 1, 8050 Zurich, Switzerland; 14 Department of Chemistry and Applied Biosciences, Institute of Pharmaceutical Sciences, ETH Zürich, Vladimir-Prelog-Weg 1-5/10, 8093 Zürich, Switzerland

**Keywords:** Mammography, Humor, Psychology

## Abstract

**Background:**

Breast cancer screening is essential in detecting breast tumors, however, the examination is stressful. In this study we analyzed whether humor enhances patient satisfaction.

**Methods:**

In this prospective randomized study 226 patients undergoing routine breast cancer screening at a single center during October 2020 to July 2021 were included. One hundred thirty-two were eligible for the study. Group 1 (66 patients) received an examination with humorous intervention, group 2 (66 patients) had a standard breast examination. In the humor group, the regular business card was replaced by a self-painted, humorous business card, which was handed to the patient at the beginning of the examination. Afterwards, patients were interviewed with a standardized questionnaire. Scores between the two study groups were compared with the Mann-Whitney U test or Fisher’s exact test. *P*-values were adjusted with the Holm’s method. Two-sided *p*-values < 0.05 were considered significant.

**Results:**

One hundred thirty-two patients, 131 female and 1 male, (mean age 59 ± 10.6 years) remained in the final study cohort. Patients in the humor group remembered the radiologist’s name better (85%/30%, *P* < .001), appreciated the final discussion with the radiologist more (4.67 ± 0.73–5;[5, 5] vs. 4.24 ± 1.1–5;[4, 5], *P* = .017), felt the radiologist was more empathetic (4.94 ± 0.24–5;[5, 5] vs.4.59 ± 0.64–5;[4, 5], *P* < .001), and rated him as a humorous doctor (4.91 ± 0.29–5;[5, 5] vs. 2.26 ± 1.43–1;[1, 4], *P* < .001). Additionally, patients in the humor group tended to experience less anxiety (*p* = 0.166) and felt the doctor was more competent (*p* = 0.094).

**Conclusion:**

Humor during routine breast examinations may improve patient-radiologist relationship because the radiologist is considered more empathetic and competent, patients recall the radiologist’s name more easily, and value the final discussion more.

**Trial registration:**

We have a general approval from our ethics committee because it is a retrospective survey, the patient lists for the doctors were anonymized and it is a qualitative study, since the clinical processes are part of the daily routine examinations and are used independently of the study. The patients have given their consent to this study and survey.

**Supplementary Information:**

The online version contains supplementary material available at 10.1186/s40644-022-00493-z.

## Background

When patients with underlying cancer are asked about the core aspects of their coping strategies in dealing with stress and fear, they frequently mention humor [1]. The positive effects of humor in medicine are widely known, but the majority of patients experience little humor or joy in the contemporary medical setting [2]. The absence of mirth can be traced back to early cultures, starting with ancient philosophers such as Plato. The trend continued in the Christian doctrine and has shaped the mode of thought in Western and European society to the present day. It also determined how a doctor should behave with patients [3]. Although MR mammography shows fantastic results, mammography in conjunction with ultrasound remains the gold standard for the detection of breast cancer [4]. Studies have shown that diagnostic exams, especially breast examinations, are extreme stressful for patients. The main stressor is the uncertainty of the diagnosis [5, 6].

Humor stimulates several physiological systems that reduce stress hormone levels, such as those of cortisol and epinephrine. Humor also enhances the activation of the mesolimbic dopaminergic reward system [7]. Furthermore, humor is one of the most effective defense mechanisms. It allows individuals to face challenges and avoid negative emotions [8]. The effectiveness of humor in reducing anxiety and increasing patient comfort in medicine, including breast examinations, has not been widely investigated so far. We found no published studies addressing the patient’s needs during radiological examinations during and after the examination.

The future role of the radiologist is a subject of ongoing discussion. Should the radiologist serve purely as an imaging expert, or should the radiologist be a patient-oriented specialist? In the latter role the radiologist would have close contact with patients and actively communicate the imaging report [9–13]. If the radiologist is in close and intensive contact with the patient, he/she should be able to establish the patient’s needs rapidly and easily. What kind of behavior would assist the radiologist in being viewed as a caring and competent doctor?

The aim of the study is to establish whether humor is a way of enhancing the patient’s comfort during breast cancer screening examinations.

## Materials and methods

### Study subjects

A prospective single-center study was conducted in accordance with the Declaration of Helsinki. Written informed consent was obtained from all patients. All patient data were anonymized, and qualitative questionnaire surveys were conducted by professionally trained interviewers 1 to 7 days after the examination. The physicians had no access to any further personal data. The examination processes and the use of different cards are part of the daily routine, regardless of the study. National regulations did not require dedicated ethics approval with anonymized lists or quality questionnaires.

Two hundred and twenty-six consecutive patients referred for routine mammography in combination with ultrasound between October 2020 and July 2021 were considered. In our institute routine breast cancer screening consists of mammography and ultrasound. The inclusion criteria were as follows: At least 18 years of age, imaging findings corresponding to the Breast Imaging Reporting and Data System (BI-RADS) 1 or 2, willingness to answer questions in a telephone interview after the examination, and successful completion of the entire telephone interview. Patients with no or insufficient knowledge of the local language and those who could not been reached within 6 days after the examination were excluded.

Patients were prospectively randomized in a one-to-one ratio prior to each examination, using a block randomization technique with blocks of six patients each.

### Study design

Prior to the examination, the participants were randomized into the “humor” or the “non-humor group”, which is part of the usual examination procedure at our institution. After the examination, the participants were contacted by phone and asked questions about their subjective experience of the examination through our quality management.

### Humor group

The doctor (AG) used the element of surprise and presented a humorously designed business card [14].

After the mammography and prior to the ultrasound examination, the participant was greeted as follows: “Good morning, my name is Dr. XY. I am your radiologist. Your mammography results were unremarkable. I realize that patients find it difficult to remember my name. For this reason, I give you my card". The situation is described in the following images more in detail (Figs. 2 and 3). The business cards handed over to the patient at the start of the conversation featured cartoon figures in this group. After this presentation, ultrasound started.

For each consultation, the time taken to perform the ultrasound examination and the dialog with the participant were measured with a stopwatch. When participants responded positively to the card, the doctor reacted spontaneously to the response. Quite often, both laughed.

The laughter or smile could not be measurably documented in the situation, since there are no technical or psychological scales for this. We can only rely on the assessment of the patients as part of the survey.

The physician had been trained in the use of humor in oncology since 3 years at a continuing education course.

### Non humor group

Participants in this group were greeted in the usual manner and given a regular formal business card (Fig. S1). There was no element of surprise. The doctor was empathetic, but professional and formal. As mentioned earlier, the routine examination procedure is conducted at our institution with and without humor. After completion of the mammography, the ultrasound examination was performed by the same procedure as in group 1. The time taken for the dialog with the participant and the ultrasound examination was measured with a stopwatch.

### Telephone interview

Between 1 and 7 days after the imaging studies, each participant received a telephone call from our professional quality management team. The interviews were conducted by two trained persons, who were blinded to the participant’s respective group affiliation. The standardized questions were developed by our quality management team. A total of nine questions were asked and the responses recorded (Table 1). The duration of the interview and its duration relative to the imaging studies were documented. The participants’ responses are summarized in Table 1.

**Table 1:** Questions and data acquired from the questionnaire

Question	Answers	Humor group (*n* = 66)	Non-humor group (*n* = 66)	*p*-value
1. Do you recall the name of your radiologist?	Yes No	Yes (*n* = 56/85%) No (*n* = 10/15%)	Yes (*n* = 20/30%) No (*n* = 46/70%)	**< 0.001**
2. How competent did the radiologist seem to you?	Scale from 1 to 5	4.91 ± 0.29 – 5; [5, 5]	4.82 ± 0.46 – 5; [5, 5]	0.094
3. How did you feel during the routine breast examination?	Scale from 1 to 5	4.86 ± 0.39 – 5; [5, 5]	4.74 ± 0.64 – 5; [5, 5]	0.247
4. If you could decide for yourself which radiology institution you would like to be examined in, would you come back to us?	1.) Yes, I would only come to you in the future. 2.) I don’t care where my gynecologist sends me. 3.) No, I’m going to a different radiology institute.	Answer 1 (*n* = 63) Answer 2 (*n* = 2) Answer 3 (*n* = 1)	Answer 1 (*n* = 65) Answer 2 (*n* = 1) Answer 3 (*n* = 0)	0.619
5. How important was the final discussion with the radiologist for you?	Scale from 1 to 5	4.67 ± 0.73 – 5; [5, 5]	4.24 ± 1.1 – 5 [4, 5]	**0.017**
6. Were you scared during your routine breast exam? (*n* = 41 for the humor group and *n* = 31 for the non-humor group)	Yes No	Yes (*n* = 7) No (*n* = 34)	Yes (*n* = 10) No (*n* = 21)	0.166
7. How satisfied were you with our radiological services in general?	Scale 1–5	4.89 ± 0.4 – 5; [5, 5]	4.86 ± 0.39 – 5; [5, 5]	0.413
8. How empathetic did the radiologist seem to you?	Scale 1–5	4.94 ± 0.24 – 5; [5, 5]	4.59 ± 0.64 – 5; [4, 5]	**< 0.001**
9. How humorous did the radiologist seem to you?	Scale 1–5	4.91 ± 0.29 – 5; [5, 5]	2.26 ± 1.43 – 1; [1, 4]	**< 0.001**

Overview of the questions and data from the questionnaire. Except for the questions asking for specific discrete answers, the data are presented as means ± standard deviation - median; [interquartile range]

### Statistical analysis

Scores between the two study groups were compared with the Mann-Whitney U test or Fisher’s exact test. *P*-values were adjusted with the Holm’s method. Two-sided *p*-values < 0.05 were considered significant. All analyses were performed by TS in the R programming language (version 4.02; R Foundation for Statistical Computing, Vienna, Austria, https://www.R-project.org/).

## Results

**Fig. 1:**
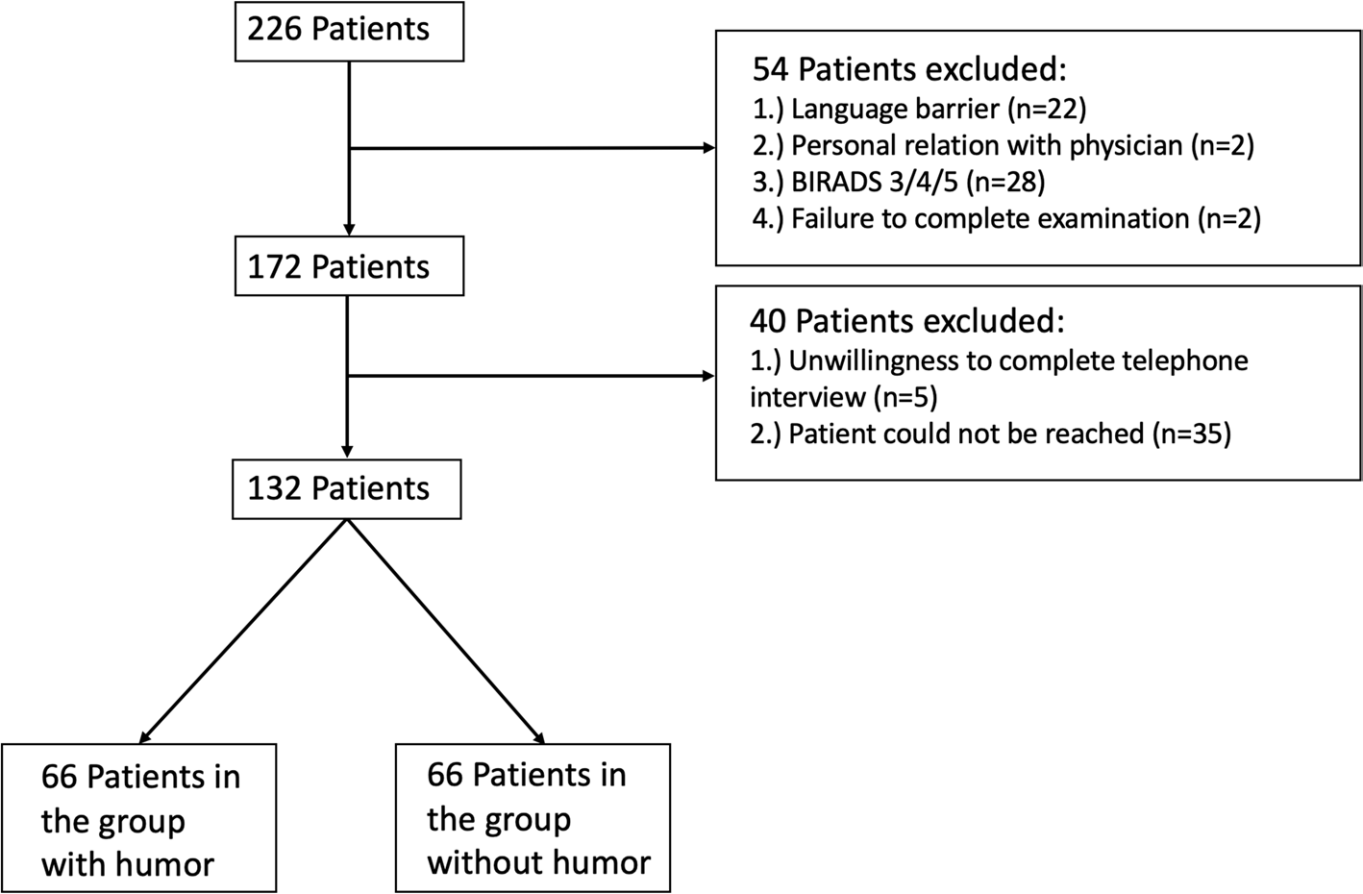
Flow diagram depicting initial number of participants and participants after exclusion criteria were implemented. Flow chart of the final study groups after the exclusion of ineligible patients

Inclusion and exclusion criteria are shown in Fig. 1.

After exclusion, 132 patients, 131 female and 1 male, (mean age 59 ± 10.6 years) remained in the final study cohort, of which 66 were in the humor group and 66 in the non-humor group (Fig. 1). The male participant was referred to our institute for a routine breast cancer examination due to his family’s medical history. The duration of the examination did not differ significantly between groups (413.3 ± 88.4 vs. 437.4 ± 76.5 sec., *p* = 0.24).

Patients in the humor group recalled the name of the radiologist more often, appreciated the final discussion (*p* < 0.05) with the radiologist to a greater extent, and felt that the radiologist was more empathetic and humorous (*p* < 0.05). While not statistically significant, patients in the humor group showed a tendency towards less anxiety (*p* = 0.166) during the examination than patients in the non-humor group. Notably, patients of both groups gave the doctor’s competence the highest rating, but professional competence was rated higher in the humor group (*p* = 0.09). In other words, physicians who used humor were considered very competent and humor did not influence the patient’s view of the physician’s competence. The results are summarized in Table 1.

## Discussion

This investigation revealed that humorous elements in communication enhance the patient’s wellbeing during routine breast cancer screening. Patients in the humor group rated the importance of the discussion with the radiologist higher (*p* < 0.05) and were able to better recall the radiologist’s name (*p* < 0.05). Patients considered a humorous doctor more empathetic (*p* < 0.05). We observed a trend towards less anxiety in patients exposed to humor during their examinations (*p* = 0.166), without reaching statistical significance. The physician’s competence was rated higher by the humor group, just below statistical significance (*p* = 0.09). There are many reasons to dispense with humor as a physician. Our data show that a physician who uses humor in communicating with the patient has no reason to be concerned about appearing incompetent.

In traditional medical settings, radiologists are invisible to patients and physicians. In one study, patients were asked about their concept of the radiologist’s task and frequently answered, “That’s the guy who always asks me if I have an allergy” [15]. Many patients are unaware of the radiologist’s role, who then remains largely invisible to patients [10].

Radiologists must be aware, that patients experience stress reactions and anxiety in a radiology department, mainly because they fear the outcome of the radiological investigation [16, 17].

Far from making the radiologist an object of ridicule, our aim is to ensure that radiologists, despite their technical expertise, can help patients feel more comfortable, improve the patient’s rating of the doctor-patient discussion, and reduce the patient’s anxiety during a radiological examination. Many studies, largely outside the medical sector, have shown that humor is an excellent coping strategy for patients. It is well known that patient’s mental wellbeing can be enhanced significantly by humor and empathy [18]. In this context, it must be mentioned that there are extremely different techniques of humor [19].

The radiologist’s role in the clinical setting is a subject of ongoing discussion, because techniques such as teleradiology or artificial intelligence will bring about major changes in the coming decades [6, 9, 12, 13, 20–31]. Should the radiologist serve exclusively as an “imager” in a dimmed room or should he/she be a patient-oriented physician in the health care management system?

Evidently, empathetic patient care is one way of showing patients that radiologists are more than persons who press buttons and ask patients if they have an allergy. Humor can be used as a means of alleviating the patient’s fear and anxiety, and assist the radiologist in leaving a stronger impression on patients than other doctors although the radiologist does not visit the patient daily at the bedside.

Humor has been a well-known factor in medicine, but research on the subject is scarce. Psychological carriers of humor are numerous. Patients usually perceive these as amusing and not ridiculous. In this context, it is interesting to note that advertisements for radiological positions often mention that people are preferred with social skills and a sense of humor can certainly be considered part of one’s social skills [32]. One of the most powerful psychological triggers of humor is surprise [33, 34].

**Fig. 2:**
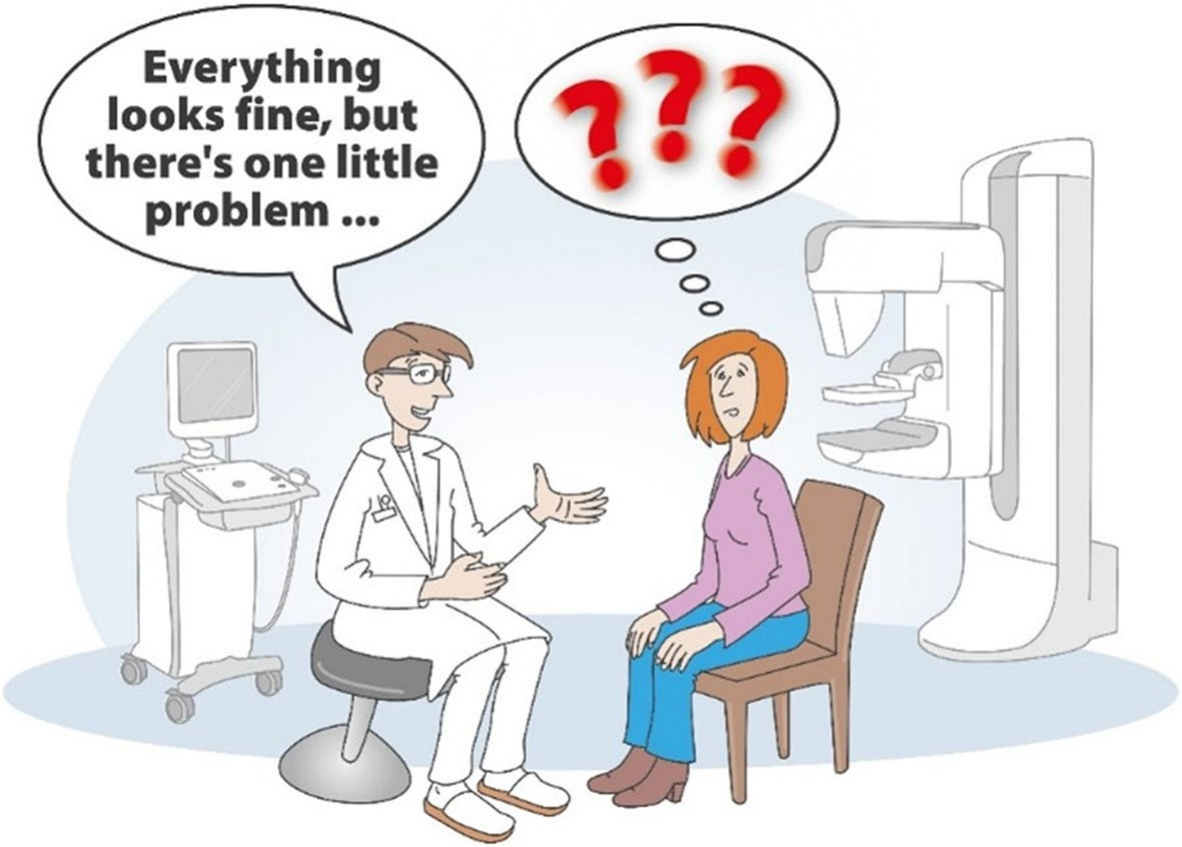
Schematic Diagram of how patients are greeted in group 1, part 1. First, the participant is told that the examination does not reveal any change. Up to this point, both groups were treated in the same manner

**Fig. 3:**
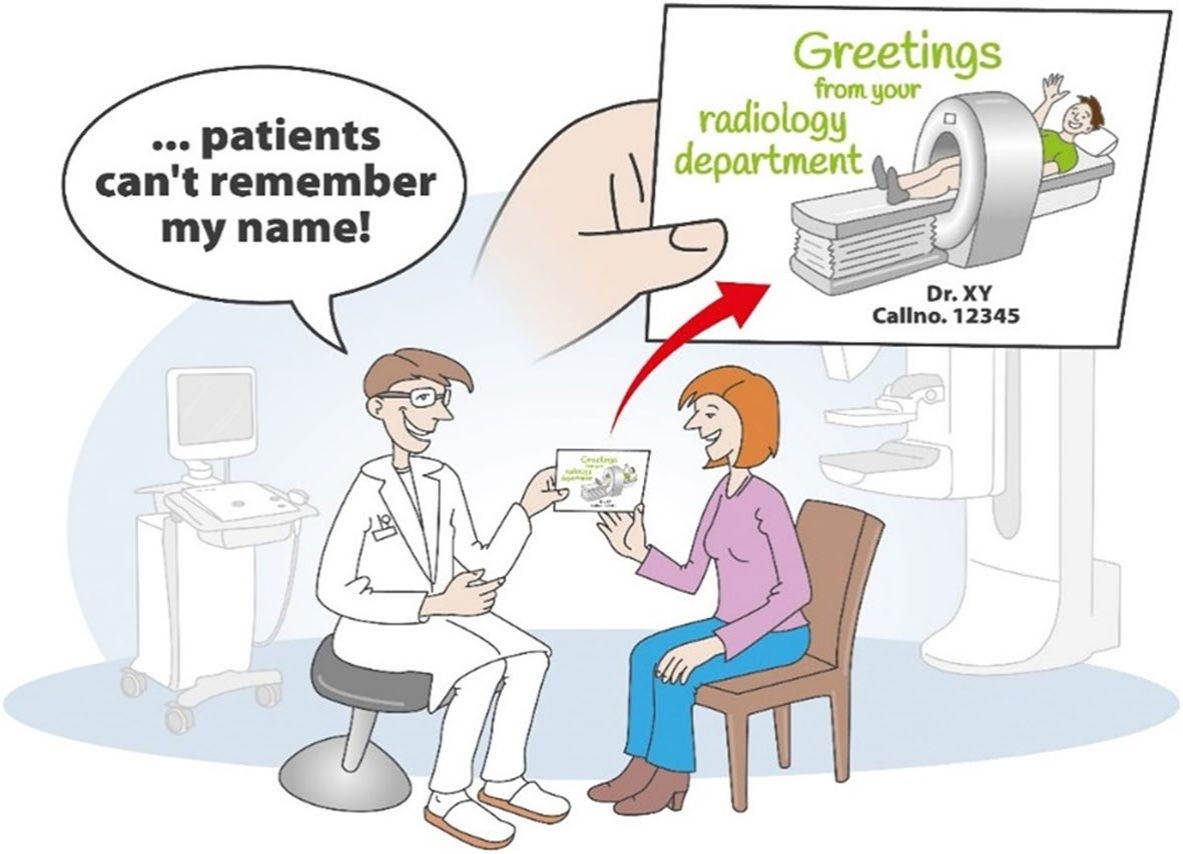
Schematic Diagram of how patients are greeted in group 1, part 2. Now the participants in group 1 receive a business card with humorous elements, but with the same information (name, academic title, phone number, mail address) as group 2. The perceivable change in the manner of greeting introduced an element of surprise, accompanied in most cases by laughter

In this investigation surprise was used when patients came in for breast center screening examinations. For decades, radiologists have entered a semi-darkened investigation room. Now, for the first time, a radiologist says, “Your examination is fine but there is one little problem: patients cannot remember the radiologist’s name.” Now the patient expects a regular business card. However, patients in group 1 (“humor”) receive a comic-like drawing on the doctor’s business card (Figs. 2 and 3). The patient experiences relief because the outcome of the investigation was no cause of concern, but is also confronted with an incongruous and surprising situation. This standardized approach was enough to lessen the patient’s anxiety, enhance wellbeing, and strengthen the doctor’s empathy.

The fact that patients are better able to recall the radiologist’s name when given a card was recently addressed in a similar study [12].

It should be noted that the practice of treating patients with and without humor is an element of our clinical routine. Therefore, the radiologists at our institution are well trained in this type of patient care. Based on the outcome of the present investigation, we recommend a similar patient care strategy at all radiology departments.

Using humor systemically without using psychological backgrounds should be used with caution. We would like to mention that in literature there are people who can react very negatively and irritated to humor. Such irritation can be caused by a clinical phenomenon called gelotophobia, that is said to affect about 5% of the population [35]. We were prepared for this, but have had consistently good experiences in the humor group in this study and in daily routine. As we adhered to the carriers of incongruity and surprise rather than jokes, we encountered no negative feedback. Only one patient in the humor group was disturbed by the telephone call, declined to give any information, and was excluded from the analysis.

This study encourages us to continue research in the field of communication and radiology. But this does not only apply to radiologists. With similar approaches, we see great opportunities to improve communication in other clinical disciplines.

This study has the following limitations. Firstly, it was a single- center study. Trials involving larger patient numbers might yield different results. As this issue concerns all medical specialties, further research is essential. Secondly, only patients with BI-RADS 1 and 2 were included. It would be interesting to investigate whether patients with more serious diseases would also respond as well. Third, humor can be expressed by various means. We used a humorous calling card. Other forms of humorous introductions could also be investigated. Fourth, human relationships are not easily standardized. Physicians have different personalities. We believe that health care professionals should be trained in the use of humor, and larger studies should be performed to determine whether humor enhances patient wellbeing. Humor can be trained and contributes to life satisfaction and health [19]. Considering the increasing rates of depression among physicians, this would be an interesting prophylaxis for the future and further research is needed concerning this topic.

## Conclusion

Humor in radiological breast cancer screening is associated with greater patient satisfaction.

Patients in the humor group remember the name of the radiologist more often, appreciated the final discussion with the radiologist to a greater extent, and felt that the radiologist was more empathetic and competent than in the non humor group.

## Supplementary Information

**Additional file 1: Figure S1.** Standard Business Card: This is the standard business card, which was distributed to participants in the non-humor group. This card is used in all departments of the hospital.

## Data Availability

The datasets used and/or analyzed during the current study are available from the corresponding author on reasonable request.

## Abbreviation

BIRADS: Breast Imaging Reporting and Data System
