# Supplementary figures and images for: Humor in radiological breast cancer screening: a way of improving patient service?
Source: Cancer Imaging. 2022 Oct 8;22:57. doi: 10.1186/s40644-022-00493-z (PMC9548186; doi:10.1186/s40644-022-00493-z)

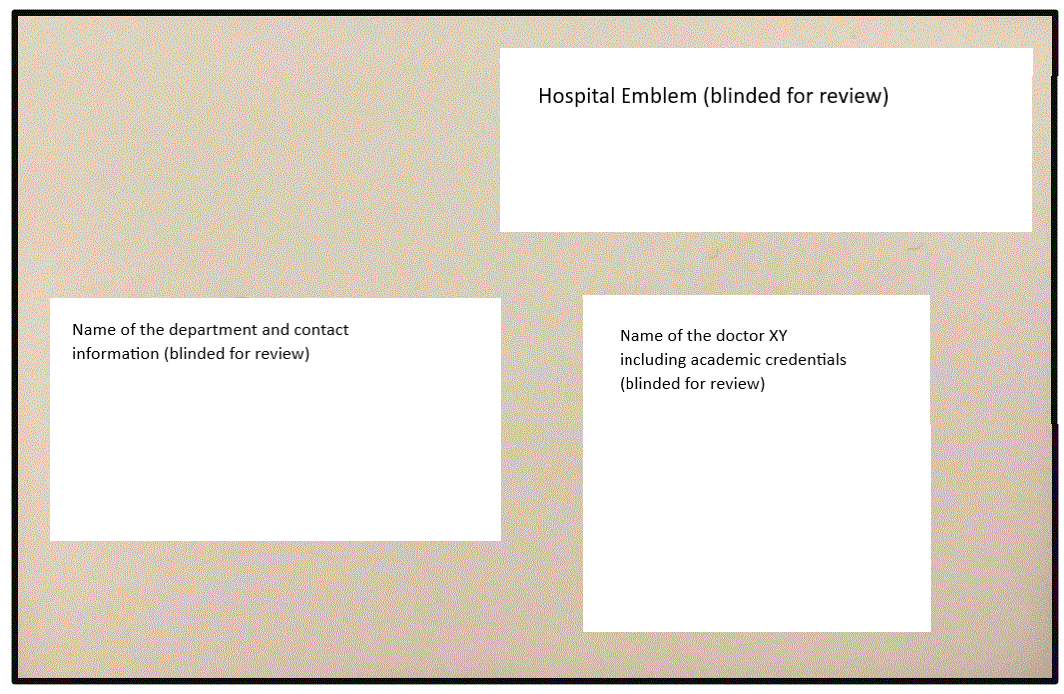

Supplement: Supplementary file 1 — Additional file 1: Figure S1. Standard Business Card: This is the standard business card, which was distributed to participants in the non-humor group. This card is used in all departments of the hospital. [file 40644_2022_493_MOESM1_ESM.tiff]
